# Anticaries potential of a fluoride foam

**DOI:** 10.1590/0103-6440202305287

**Published:** 2023-03-06

**Authors:** Larissa Caroliny de Brito Benedito, Antônio Pedro, Cinthia Pereira Machado Tabchoury, Jaime Aparecido Cury

**Affiliations:** 1 Department of Biosciences, Piracicaba Dental School, UNICAMP, Piracicaba, SP, Brazil

**Keywords:** fluoride, calcium fluoride, sodium fluoride, topical fluoride, tooth enamel

## Abstract

Foam has been used worldwide as a vehicle for the professional application of fluoride and hypothetically should have the same anticaries potential as conventional fluoride gel (F-gel) in terms of the formation of reaction products with enamel. Thus, the ability of Flúor Care^®^ foam (FGM, Joinville, SC, Brazil, 12,300 ppm F, acidulated) to react with enamel was evaluated in comparison with Flúor gel^®^ (DFL, Rio de Janeiro, RJ, Brazil, 12,300 ppm F, acidulated). Slabs (n=10/group) of sound enamel and with caries lesion were used, in which the concentrations of total fluoride (TF), and loosely (CaF_2_-like) and firmly (FAp) bound types were determined. The importance of agitation during application was previously tested. The determinations were made with fluoride ion-specific electrode and the results were expressed in μg F/cm² of the treated enamel area. ANOVA and Tukey tests were used to analyze the difference among treatments, independently for sound and carious enamel. The agitation of the products during application significantly increased the reactivity of the foam (p<0.05), but not that of the gel (p>0.05). The foam did not differ from F-gel (p>0.05) concerning the formation of TF and CaF_2_-like in sound or carious enamel. Regarding FAp, the foam did not differ from F-gel (p>0.05) in the carious enamel, but the concentration in the sound was lower (p<0.05). The results show that this commercial fluoride foam tested needs to be agitated during application to improve its reactivity with enamel, which raises a question about other brands.

## Introduction

The professional fluoride application (PFA) for controlling dental caries has been used for over 50 years, using products of high fluoride concentrations (9,000 to 54,500 ppm F). In the past, aqueous solution of NaF at 2% was used, but currently, commercially available PFA products are formulated in gel, foam and varnish form. These different formulations were developed and launched on the market for some reason. Particularly, in the case of fluoride foam, it was created as a safer alternative than PFA gel (F-gel) to reduce fluoride intake by children ^1^ ^,^ ^2^. In addition to the safety factor in the clinical recommendation of the various vehicles for PFA, the anticaries potential of these commercial products should be evaluated and tested before commercialization ^3^. The effect of pre-procedures, as well as those during and after the application of products, should be systematically evaluated.

The potential of the anticaries efficacy of vehicles for PFA can be estimated in the laboratory by the reactivity of fluoride with dental enamel, whether sound (to estimate the "preventive" effect) or with caries lesion (carious, to assess the "therapeutic" effect). Due to the high concentration of fluoride that is applied to enamel, the result is the formation of a high concentration of chemical reaction product. The total fluoride (TF) formed can be differentiated into two reaction by-products ^4^, one type is calcium fluoride (CaF_2_-like), and the other type is fluorapatite (FAp). These can be differentiated because the CaF_2_-like is soluble in alkali, being extracted first with KOH, and the FAp, being insoluble, is then extracted with acid ^5^. In addition, CaF_2_-like formed in enamel in a greater quantity than FAp ^4^, has been considered, in terms of the anticaries potential, as the most important by-product ^5^. Given the importance of the chemical reaction with enamel in the formation of reaction products, the effect of drying or not the dental surface before application, agitation, and time during application, and washing or not washing the tooth after the application must be evaluated. However, the factors that interfere with the reactivity of fluoride with enamel have been extensively evaluated for gel (6, among others) and varnish (7, among others), but not for the anticaries potential of foam. In addition, the mechanism by which the reactivity of the fluoride of the varnish with the enamel occurs differs from the gel and foam ^8^. Gel and foam, besides presenting the same fluoride concentration and pH, are comparable in terms of reaction mechanism with enamel. In addition, the anticaries efficacy of F-gel is evidence-based ^9^ and thus it can be used as a positive control for the anticaries potential of foam.

Therefore, Wei and Hattab ^1^ compared the reactivity of acidulated foam with the gel when applied in vitro to the enamel for 4 min at room temperature and 100% humidity. After application, the dental surfaces were washed with water for 30 s, followed by washing with deionized water for 1 min. The authors did not observe a statistically significant difference between foam and gel in the TF concentration found at 5 μm from the enamel surface, however, the foam was less reactive at higher depths. Whitford et al. ^10^ compared in vivo the difference between foam and F-gel in terms of reactivity with enamel. The applications were made for 4 min with trays and the products did not differ in terms of TF concentrations formed. Hayacibara et al. ^11^ found no statistical difference between acidulated foam and F-gel in the formation of CaF_2_-like in sound enamel. Applications were made with swabs for 4 min, however there are no details if there was agitation of the products during the application. More recently, Delbem et al. ^12^ compared foam with neutral F-gel in terms of the formation of CaF_2_-like and FAp in enamel after application for 1 min and found no significant difference between the products. This research showed that washing the enamel with water shortly after application does not reduce the concentration of products formed by the application of foam as of gel, corroborating a previous study done with F-gel ^13^.

As reported above, although studies have been made to evaluate the anticaries potential of fluoridated foam, none of the studies mentioned was systematized to evaluate the importance of the enamel surface being dry at the time of application, as well as the effect of agitation during application. Furthermore, it is also not known if the foam would not form the same concentration of reaction products in enamel in 1 min as in 4 min. Likewise, the importance of time of water rinsing the enamel after application has not been systematically evaluated. In addition, although the anticaries efficacy of foam has already been clinically evaluated, there is still no strong evidence for its recommendation ^14^.

Thus, the present study aimed to assess the anticaries potential of fluoridated foam compared to F-gel, in terms of reactivity with enamel sound and with caries lesion, using a standardized protocol considering the abovementioned variables. We hypothesized that foam with fluoride concentration and pH similar to F-gel, would have equivalent anticaries potential compared with F-gel in terms of reaction products formed in enamel.

## Materials and methods

### Experimental design

This study evaluated whether acidulated fluoridated foam would have the same anticaries potential as fluoride gel (F-gel). Three bottles of Flúor Care^®^ foam (FGM, 12,300 ppm F, acidulated) were purchased at different places of sale. As a positive control, acidulated Flúor gel^®^ from DFL (12,300 ppm F) was purchased and used. All products were within the shelf life declared on the packaging. The fluoride concentration in the products was determined by the direct technique with an ion-specific electrode for fluoride (ISE-F). As the concentrations found were in accordance with the expected, one bottle of foam was randomly chosen for the reactivity test with the enamel.

**Figure 1:**
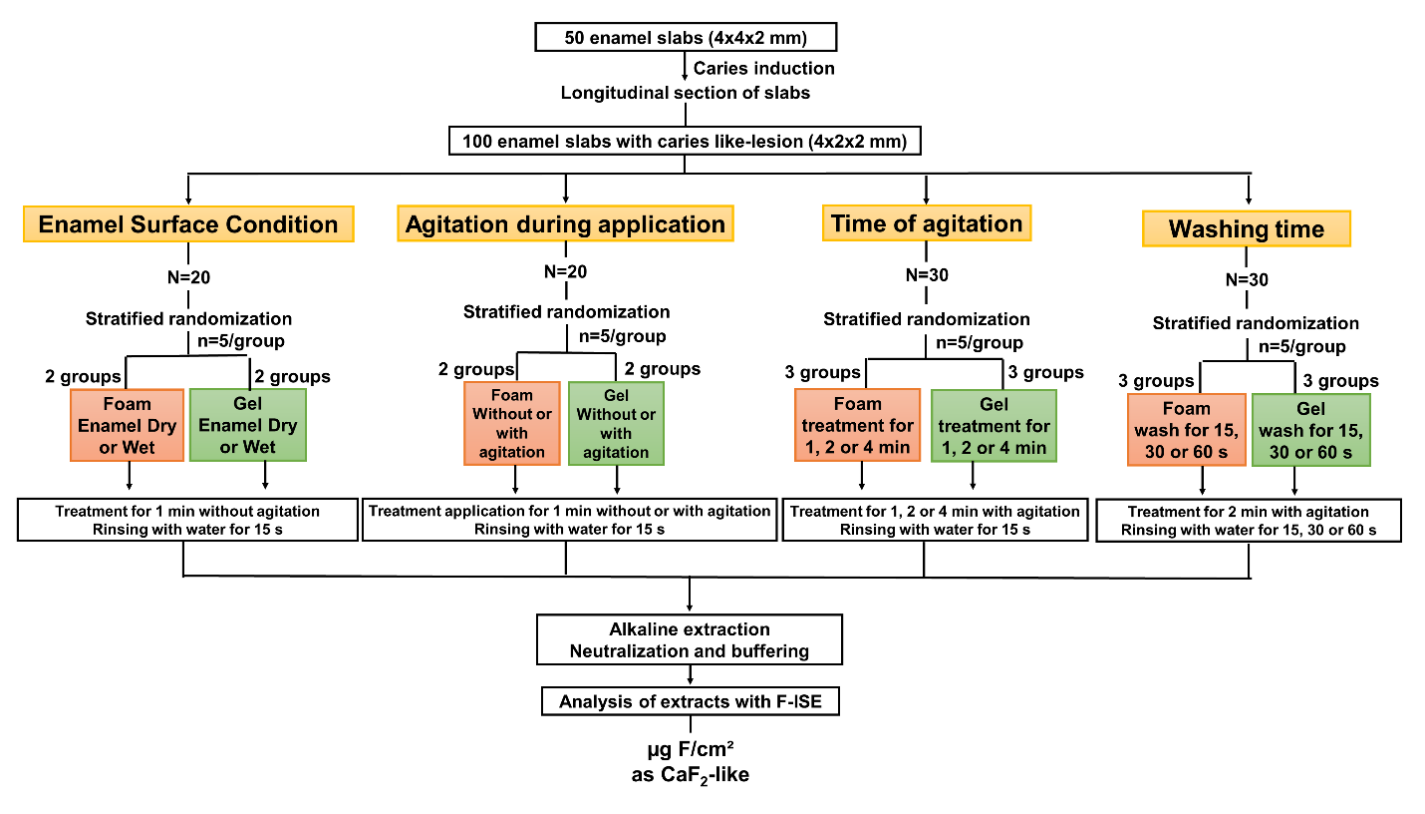
Flowchart of the experimental design for the standardization of the reactivity protocol

An in vitro, randomized, treatment-related and paired study was conducted in relation to reactivity products formed in sound enamel and with caries lesions. The experiment was conducted in two steps. In the first (Figure 1), enamel slabs with induced caries lesion (n=5/variable) were used. The slabs were treated with foam or F-gel, being evaluated the importance of the following parameters in terms of the concentration of fluoride type CaF_2_ (CaF_2_-like) formed: i) being the surface of dry enamel; (ii) agitation during application; iii) duration of agitation and iv) the rinsing time of the slab after application.

**Figure 2:**
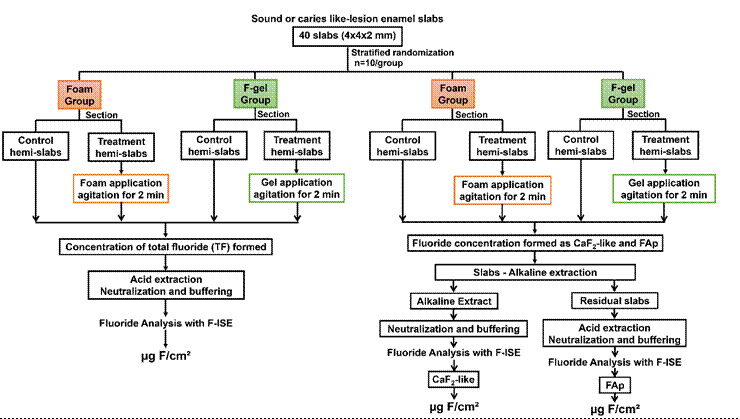
Flowchart of the experimental design to compare the reactivity of the foam with the F-gel in the formation of TF, CaF_2_-like, and FAp in the sound and carious enamel.

In the second step (Figure 2), slabs (n=10/group) of bovine sound enamel and with induced caries lesions were stratified between the treatments with foam and F-gel, based on surface hardness. The slabs were sectioned in half; one half of each slab was treated with foam or F-gel for 2 min under agitation and the other was used as a negative control (baseline), characterizing a paired design. The concentrations of total fluoride (TF), in the form of CaF_2_-like and fluorapatite type (FAp), were determined in the treated and control halves of each slab. The results found in the treated hemi-slabs were subtracted from the controls and expressed in μg F/cm². The results of the standardization and the final reactivity were analyzed by ANOVA followed by the Tukey test (α=5%), independently for the sound and carious enamel, and for TF, CaF_2_-like and FAp formed.

The hypothesis of this study was that foam, because of similar fluoride concentration and pH to F-gel, would have the same anticaries potential in terms of reaction products formed in sound or carious enamel.

### Determination of fluoride and pH of foams and gel

Of each bottle, an amount of 100.0 mg (±0.01) was weighed in duplicate, which was dissolved in ultrapure water and the final volume was completed to 100 mL. Duplicates of 1.0 mL of each extract were buffered with an equal volume of TISAB II (1.0 M acetate buffer, pH 5.0, containing 1 M NaCl and 0.4% CDTA) and fluoride concentration was determined with ISE-F, as described in the "fluoride analysis" section. The pH was estimated with indicator paper (MQuant®, Merck KGaA, Darmstadt, Germany; lot number: HC178067) (±0.5 pH units).

### Preparation of enamel slabs and induction of caries lesion

Enamel slabs (4x4x2 mm) were obtained from bovine incisor teeth and their surfaces were flattened and polished ^15^. Surface hardness (SH) was determined and 200 slabs with SH of 331.9±17.3 kg/mm^2^ (mean±SD) were selected. One hundred and sixty slabs were immersed (2 mL of solution per mm² of enamel surface area) in 0.1 M acetate buffer pH 5.0 containing 1.28 mM Ca, 0.74 mM Pi and 0.03 μg F/mL, for 12 h at 37°C, to induce subsurface caries lesion ^16^. SH was again determined and 90 slabs with hardness of 9.6±4.8 Kg/mm² were selected for the standardization of foam and F-gel application conditions and for the final reactivity test comparing these professional application products. To standardize the methodology of application of the products (Figure 1), 50 carious slabs were longitudinally sectioned, resulting in 100 slabs (2x4x2 mm) that were distributed in a stratified way, based on their surface hardness, for the standardization variables (n=5/variable). For the analyses of TF, CaF_2_-like and FAp (Figure 2), the remaining 40 demineralized slabs and 40 sound slabs were stratified between treatments (n=10/group), also based on their surface hardness. The length and width of the hemi-slabs were determined with a digital caliper (± 0.01 mm) to calculate the enamel area that was exposed to the treatments. All other surfaces of the hemi-slabs, except for the enamel surface, were then protected with wax 7.

### Standardization of the methodology of application of products

For standardization, carious enamel slabs were used, and the effect of the variables studied was estimated in terms of the concentration of fluoride-type CaF_2_-like formed in enamel. The flowchart in Figure 1 illustrates the evaluated parameters, the details of which are described below:

### i) Importance of enamel surface being dry

Twenty slabs were distributed in 4 groups of 5 to evaluate the importance of the dry enamel surface for the chemical reaction of fluoride from F-gel or foam with enamel (Figure 1). Drying was done for 15 s with air jet. Enamel moistening was done by distributing 3 drops of 1 μL of ultrapure water across the surface of each slab right before applying the products. A quantity of foam or gel (~0.4 g/slab) was placed on the enamel surface of each slab using one cotton swab per application. The products were not stirred and after 1 min the slabs were washed for 15 s with ultrapure water jets.

### (ii) Agitation or not of the product during application

Twenty slabs were distributed in 4 groups of 5 to evaluate the effect of foam and F-gel agitation on fluoride reaction with enamel (Figure 1). The surface of the enamel of each slab was moistened as already described. With the aid of a cotton swab, approximately 0.4 g of foam or gel was deposited on the enamel surface. The agitation was made for 1 min with the swab itself, performing circular movements every second. After 1 min of agitation or not, the slabs were washed for 15 s with ultrapure water jets.

### iii) Agitation time during the application

Thirty slabs were distributed in 6 groups of 5 to evaluate the effect of the agitation time of F-gel or foam applied on the fluoride reaction with enamel (Figure 1). The approximate amount of 0.4 g of foam or gel was applied on the surface of the moistened enamel of each block, with a cotton swab. Agitation was done for 1, 2, or 4 min and the slabs were washed for 15 s with ultrapure water jets.

### iv) Duration of water rinsing after application

Thirty slabs were distributed in 6 groups of 5 to evaluate the effect of the duration of water rinsing after application of F-gel or foam, either in terms of removal of the applied products or on the reduction of the concentration of reaction products formed in enamel (Figure 1). The application was made as described in the previous item, fixing the agitation time in 2 min. The rinsing times tested were 15, 30, and 60 s. The fluoride analysis protocol of type CaF_2_-like formed in enamel is described in the session "Determination of CaF_2_-like and FAp formed".

### Reactivity of foam and gel with enamel

The flowchart in Figure 2 illustrates the procedures performed, the details of which are described below: The enamel surface was moistened and with the aid of a cotton swab, approximately 0.4 g amount of foam or F-gel was applied. The applied products were agitated for 2 min with circular movements and then washed for 60 s with ultrapure water jets. The control hemi-slabs were treated with ultrapure water.

### Determination of total fluoride (TF) formed in enamel

The TF formed in the enamel was extracted with acid by the serial removal of 3 layers of enamel, as previously described ^13^. Each slab was individually placed in a first test tube, to which 0.25 mL of 0.5 M HCl was added, and after 15 s under agitation, the slab was removed, washed for 30 s with ultrapure water and transferred to another tube. This extraction was repeated using two more tubes, but by the times of 30 and 60 s of agitation. To extracts, 0.25 mL of TISAB II (containing 0.5 M NaOH) was added and fluoride in these solutions was determined with ISE-F as described in the session "Fluoride analysis". The amounts (μg) of fluoride found in each extract were summed; the value divided by the treated enamel area, and the result was expressed in μg F/cm^2^. The result found in the enamel of the treated hemi-slab was subtracted from the existing one found in the respective control hemi-slab, and thus the net result is TF formed by the treatment done.

### Determination of CaF_2_-like and FAp formed in enamel

As illustrated in Figure 2, the concentrations of CaF_2_-like and FAp formed were sequentially determined in the enamel of the same slab, first extracting with alkali the loosely bound fluoride, CaF_2_ type (CaF_2_-like), followed by extraction with acid of the firmly bound fluoride residual, not soluble in alkali, a previously described methodology ^5^.

Each enamel slab was placed in a test tube, to which 0.25 mL of 1 M KOH was added. The slab was removed, washed, and transferred to another tube for acid extraction and determination of FAp concentration, according to the methodology described for TF.

Fluoride concentration in alkaline and acid extracts was determined with ISE-F, as described in the "Fluoride analysis" section. The amount (μg) of fluoride found in the alkaline extract was divided by the area of the treated enamel, and the result was expressed in μg F type CaF_2_-like per enamel area (μg F/cm^2^). The FAp concentration was also expressed in μg F/cm^2^, as described for TF. The result found in the enamel of each treated hemi-slab was subtracted from the existing one found in the respective hemi-control slab, and thus the net result represents CaF_2_-like and FAp formed by the treatment made.

### Fluoride analysis with ion-specific electrode

All determinations were made with ISE-F Orion 96-09 (Thermo Scientific Orion, Boston, MA, USA) coupled to a VersaStar (Thermo Scientific Orion) ion analyzer, calibrated according to each determination made, as described:

To determine the fluoride concentration in foams and gel, the equipment was calibrated with standards containing 0.5 to 16 μg F/mL and TISAB II at 50%. For the determination of CaF_2_-like, calibration was performed with standards containing 0.03 to 16 μg F/mL, 0.5 M KOH, and TISAB II (containing 0.5 M HCl) at 50% (v/v). For the determination of TF and FAp, calibration was performed with standards containing 0.03 to 16 μg F/mL, 0.25 M HCl, and TISAB II (0.5 M NaOH) at 50% (v/v). All fluoride standards were prepared with NaF 99.99% (Sigma-Aldrich, lot 215309, St Louis, MO, USA). The linear regression coefficient between the fluoride concentrations of the standards and the respective mV values were calculated using the Excel spreadsheet^®^ (Microsoft Corporation., Chicago. USA). The r^2^ values of all curves were at least 0.999. The accuracy of the calibration curves was verified with a standard Orion fluoride solution 940907 (Thermo Fisher Scientific Inc.) and the percentage of variation between the found and the expected ranged from -0.71 to 2.0%.

The mV values of the sample readings were converted to fluoride concentration using the same Excel worksheet^®^ as the calibration. The results of fluoride concentration found in the foam and gel used were the amount of fluoride by weight (mg F/kg). The liquid concentrations of fluoride-formed enamel (TF, CaF_2_-like and FAp) were expressed as the amount of fluoride per treated area (μg F/cm²).

### Statistical analysis

The Shapiro-Wilk test evaluated the normality of the error distribution. The concentrations of TF, CaF_2_-like and FAp were analyzed by ANOVA followed by the Tukey test (α=5%). The standardization data of the methodology were independently analyzed for foam and gel. The comparison between foam and gel in terms of TF, CaF_2_-like, and FAp formed in enamel was made independently for the sound and the carious enamel. All analyses were performed by the SPSS Statistics 26.0 application (IBM Corporation, New York, USA).

## Results

The fluoride concentration in the two products was similar to that reported by the manufacturers, 12,193 and 12,167 μg F/g, respectively for foam and gel. It was also confirmed that the products were acidified (pH ~3.5 for both products).

**Figure 3:**
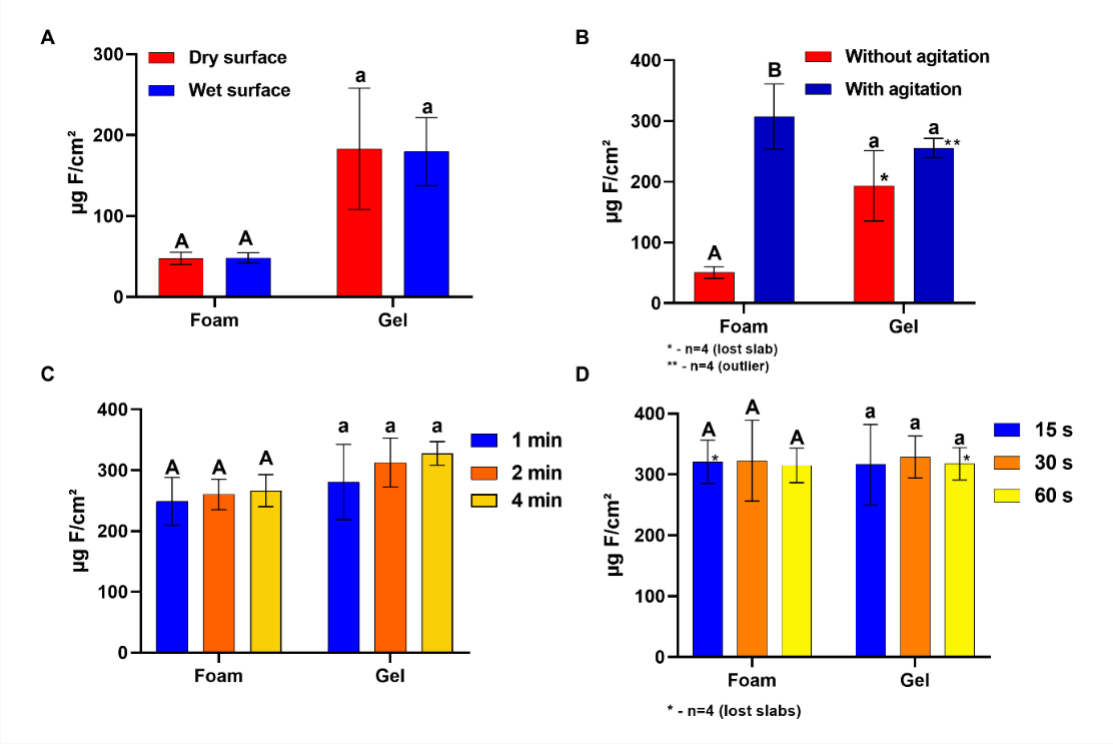
Concentration (μg F/cm²) of type CaF_2_-like found in enamel (mean±SD;n=5) by the reaction with the foam and F-gel used, according to the tested variables: (A) Dry or wet enamel; (B) agitation during application; (C) agitation time and (D) rinsing time. Distinct letters denote statistically significant differences between the variables (p<0.05), independently, for foam (uppercase letters) and gel (lowercase).

Figure 3 shows that, among the variables tested that could interfere with fluoride reactivity with enamel, product agitation during application (Figure 3B) was the only relevant variable. When foam was agitated during application, the reactivity increased from 50.9 to 307.4 μg F/cm² and the difference was statistically significant; for gel, the effect of agitation was not statistically significant (p>0.05).

**Figure 4:**
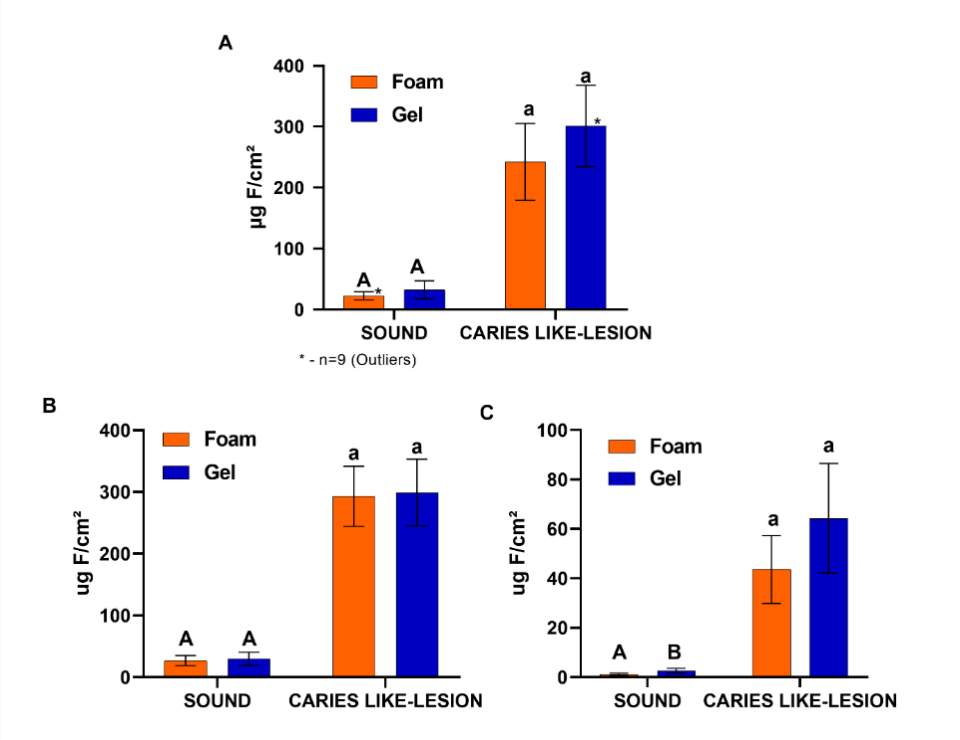
Concentration (μg F/cm²) of TF (A), CaF_2_-like (B), and FAp (C) formed in sound and carious enamel (mean;SD;n=10) after treatments with the foam or gel used. Distinct letters denote statistically significant differences between foam and gel (p<0.05), independently, for sound enamel (uppercase letters) and carious (lowercase).

Figure 4 shows that the foam did not differ from the F-gel in relation to the formation of TF (Figure 4A) and CaF_2_-like (Figure 4B), either in reactivity with sound or carious enamel (p>0.05). In terms of FAp formed (Figure 4C), the foam did not differ from the F-gel for carious enamel (p>0.05), but the concentration in sound was lower (p<0.05).

## Discussion

The data obtained supported the hypothesis of this study, in the sense that the foam having the same pH and the same fluoride concentration would have the same anticaries potential as F-gel in terms of reactivity with dental enamel. However, this premise is only valid if the foam is agitated during its application on the surface of the enamel, and to the best of our knowledge, the finding is being reported for the first time. In addition, the effect of factors involved in the pre-, during, and post-application, whether foam or F-gel in enamel, have never been systematically evaluated as done in the standardization of the reactivity methodology used in the present study.

For the standardization of the reactivity methodology, we chose to use enamel with caries lesion and to evaluate the effect of the variables studied by the concentration of CaF_2_-like formed by two reasons. First all because CaF_2_-like is considered the reaction byproduct formed in enamel responsible for the anticaries mechanism of action of fluoride for professional use ^5^ ^,^ ^17^. Secondly, because greater CaF_2_-like concentration is formed in the carious enamel than in the sound ^18^, increasing the sensitivity of finding small difference of the effect of factors under study. The first factor evaluated in the present study was the importance of the enamel surface being dry before fluoride application. Our results showed (Figure 3A) that there was no significant difference in the formation of CaF_2_-like in wet or dry enamel, either by the application of foam or F-gel. The importance of being dry enamel before professional fluoride application has clinical implication and has been recommended in the past when aqueous solution of NaF 2% was used for direct application on dental surfaces ^19^. At the present, gel, foam and varnish have been used as vehicles for professional fluoride application and the relevance of the dental surface being dry has to be discussed for each case. For varnish application, the surface should be dry to allow the adhesion of the varnish on the applied surface and the subsequent time-dependent fluoride reactivity ^7^ ^,^ ^8^ ^,^ ^20^ ^,^ ^21^. However, the relevance of this factor for the reactivity of foam and gel fluoride is totally distinct. Unlike varnish, both foam and gel are products of the immediate reaction of fluoride with enamel, and the understanding of how the chemical reaction occurs should be considered ^4^ in the present discussion. The reactivity of fluoride with enamel depends directly on the fluoride concentration of the applied product and the availability of calcium from enamel ^22^. The fluoride concentrations of both foam and gel are maintained during the reaction with enamel because they are applied to the teeth with trays, not having during the reaction dilution by saliva, different from what occurs with the application of NaF solution at 2%. Thus, the most important factor to be considered is the availability of calcium for the chemical reaction. During the reaction, the aqueous liquid medium is important not only to provide calcium from the tooth to the hydration layer of the enamel, as for the fluoride of the product to be in ionic form, react with calcium, and form CaF_2_-like. Both foam and gel are aqueous media, which means that fluoride is in its free ionic form and calcium is naturally present in the hydration layer of enamel ^23^. The limiting factor in the reaction is the amount of calcium that is increased by the acid pH of the foam and gel applied. Thus, the result found of the non-effect of drying the enamel surface in terms of reactivity of the fluoride of the applied foam or gel is explainable.

The second factor evaluated was the importance of product agitation during application. It was decided to moisten the enamel surface because during the clinical application of the foam using a tray, there will always be a film of saliva on the teeth ^24^, even if they were "dried" with an air jet. The results found (Figure 3B) showed that agitation was fundamental to increase the reactivity of foam fluoride, but not that of gel. To our knowledge, this information has never been reported before, for which there must be an explanation. The most likely explanation of the difference between foam and gel is physical and non-chemical in nature, as discussed in the previous paragraph. In both foam and gel, fluoride is in free ionic form to react with the enamel, but the air bubbles present in the foam limit the interaction of fluoride with the enamel surface. Thus, the agitation of the foam during application should increase the surface of contact with the enamel, enhancing its reactivity. The result found may have clinical relevance regarding the anticaries potential of fluoridated foam in terms of formed reaction products. Foam has been applied with double trays and the only clinical recommendation is that the patient should be occluding ^10^. Most likely, reactivity will depend on the patient's behavior in terms of occlusal movements during application and should be further evaluated. On the other hand, the need for agitation was observed for this specific commercial brand of foam.

Although agitation has been shown to be important only for the reactivity of fluoride foam, it was also done during the application of the gel when evaluating the effect of the agitation time factor. Reasonable clinical times of application (1, 2 and 4 min) were simulated, and our results showed that the effect of reactivity was not time dependent (Figure 3C), either for foam or gel. This result was already known for F-gel from studies done in vitro ^25^ and in situ ^6^ ^,^ ^26^. The absence of effect of time is explainable because the chemical reaction between fluoride and enamel is self-limiting, mainly because the products used were acidic. The reaction is instantaneous but is limited by the amount of calcium for the reaction, not the fluoride, which is in excess either in the foam as in the gel. As the diffusion of acid to remove more calcium from the inside of the enamel is self-limited, the reactivity reaction reaches equilibrium in a short time. It should be emphasized the clinical relevance of the present data because if the time of application of the foam could be reduced from 4 to 1 min ^27^, among others) without impairing the anticaries efficacy of foam application, its safety in terms of fluoride intake during application would increase.

Regarding the standardization test of the importance of time of rinsing enamel with water after product application, this issue should be discussed from either an experimental point of view as well as its clinical relevance. Our results showed (Figure 3D) that the enamel, with the products applied on it, can be rinsed with water for up to 1 min, without reducing the effect of fluoride reactivity. This result is important in the laboratory to avoid further contamination in the fluoride analysis. Thus, residuals of foam or gel adhered to the enamel surface would be washed away. This result is also of clinical relevance and was already known for F-gel that rinsing enamel with waterjet does not reduce the concentration of reaction products formed ^13^, which is also valid for foam ^12^. The greatest concern would be the dissolution of the weakly bound fluoride formed, but in addition to pure CaF_2_ being a low solubility salt in water (1.5 mg), the solubility of the CaF_2_-like formed is even lower ^28^. Thus, washing the teeth with water jets after applying fluoridated foam would increase the safety of fluoride intake from the foam that is adhered not only to the teeth but also throughout the oral cavity. In summary, the results of the present study (Figure 4, A, B) clearly showed that in terms of reactivity, acidulated foam has the same anticaries potential as acidic F-gel, however it needs to be shaken during application (Figure 3B). The equivalence of the anticaries potential was compared with F-gel, not only by the similarity of clinical application, but also by the fact that the anticaries efficacy of acidulate F-gel is based on evidence ^9^. This equivalence was estimated by the formation of CaF_2_-like (Figure 4B), the reaction byproduct considered responsible for the anticaries effect of fluoride and professional use ^5^. It has also been demonstrated that this equivalence with F-gel is valid for sound enamel and with caries lesion (Figure 4 B). Thus, it is expected that the foam would have the same anticaries efficacy, from a preventive point of view to interfere with the development of caries when applied in sound enamel, as therapeutic in the inhibition/reversal of pre-existing caries lesion in enamel. In addition, the results of CaF_2_-like formed (Figure 4 B) were equivalent to those of TF (Figure 4A), suggesting that the determination of TF can be made instead of CaF_2_-like to estimate the anticaries potential of professional fluoride. The advantage of determining TF rather than CaF_2_-like is that it would determine how deep in the enamel surface the chemical reaction occurred. In the present work, this was made, and the reactivity extended up to 100 μm from the anatomical surface of the enamel but did not differentiate the F-gel foam (data not shown).

This laboratory study, which evaluated the anticaries potential of foam used for professional fluoride application, was done not only in conditions simulating the clinical use of fluoride but also compared with F-gel, used as a positive control because there is evidence of its anticaries efficacy, however, it has limitations. Thus, the importance of foam agitation during application should be clinically evaluated with the use of trays and not swabs agitation, because the application of foam is usually not done individually on an isolated dental surface. However, the need for agitation was observed for the commercial FGM product analyzed and thus other foam trademarks should be tested to rule out that this is not an inherent problem of the product used. In addition, the anticaries potential of the foam was estimated by the concentration of reaction products formed in enamel. Although there are high correlations between CaF_2_-like formed in enamel, the release of fluoride ion to biofilm fluid, and the consequent reduction of enamel demineralization submitted to the cariogenic challenge, further studies need to be done for this evaluation.

In conclusion, the results show that this commercial fluoride foam tested needs to be agitated during application to improve its reactivity with enamel, which raises a question about other brands.
